# Association of the rs1424954 polymorphism of the ACVR2A gene with the risk of pre-eclampsia is not replicated in a Finnish study population

**DOI:** 10.1186/1756-0500-4-545

**Published:** 2011-12-19

**Authors:** A Inkeri Lokki, Miira M Klemetti, Sanna Heino, Leena Hiltunen, Seppo Heinonen, Hannele Laivuori

**Affiliations:** 1 Department of Medical Genetics, Haartman Institute, University of Helsinki, P.O. Box 63 (Haartmaninkatu 8), FI-00014 Helsinki, Finland; 2 Women's Health, Research Programs Unit, University of Helsinki, Helsinki, Finland; 3 Department of Obstetrics and Gynaecology, Helsinki University Central Hospital, Helsinki, Finland; 4 The National Graduate School of Clinical Investigation, University of Helsinki, Helsinki, Finland; 5 Department of Haemostasis, Finnish Red Cross Blood Service, Helsinki, Finland; 6 Department of Obstetrics and Gynaecology, Kuopio University Hospital, Kuopio, Finland; 7 University of Eastern Finland, Kuopio, Finland

**Keywords:** ACVR2A, Pre-eclampsia, SNP, Activin A

## Abstract

**Background:**

Pre-eclampsia/eclampsia is a common vascular pregnancy disorder associated with high maternal and infant mortality and morbidity worldwide. The role of Activin A and more recently type 2 Activin A receptor (ACVR2A) in the pathogenesis of pre-eclampsia has been the subject of genetic and biochemical research with controversial results.

**Findings:**

We genotyped a candidate pre-eclampsia-associated single nucleotide polymorphism rs1424954 in *ACVR2A *in three independent study populations of Finnish pre-eclamptic (total N = 485) and non-pre-eclamptic (total N = 449) women using pre-designed TaqMan allele discrimination assay and polymerase chain reaction. The possible association of the alleles and genotypes of interest with pre-eclampsia was evaluated using the chi-square test and logistic regression analysis. We found no association of rs1424954 to pre-eclampsia in Finnish patients.

**Conclusions:**

rs1424954 was not associated to pre-eclampsia in the Finnish study population. We hypothesise that while the gene associates to pre-eclampsia worldwide, the causative polymorphism in *ACVR2A *may be unique in genetically differing populations. Further research is needed to characterise the haplotype structure of *ACVR2A *in order for the causative genetic variant to be identified.

## Background

Pre-eclampsia (PE) is a serious disorder of human pregnancy characterised by new-onset hypertension and proteinuria after 20 weeks of gestation. PE may lead to multi-organ dysfunction and rarely, to a life-threatening convulsive condition, eclampsia (E). PE affects 3-5% of pregnancies worldwide and occurs in all ethnic groups. It is considered a feto-maternal problem, immunology-related poor placentation playing a role in the etiology [1], The development and the progression of the disease are unpredictable, and no effective therapy is currently available apart from the delivery of the placenta [2]. In the mother, PE is associated with an increased risk of cardiovascular disease (CVD) in later life [2,3].

The cell signaling protein activin A is a dimeric member of the transforming growth factor-β (TGF-β) superfamily of signaling proteins. The role of TGF-β proteins has previously been described in various cellular processes required for reproductive function including regulation of cell proliferation, differentiation and apoptosis [4,5]. Activin A plays a role in endothelial regulation and maternal endovascular inflammatory response [6-8]. Activin receptors type I and type II, hereafter ACVR1 and ACVR2, are barrel like transmembrane receptor proteins. Activin A binds with ACVR2 which associates with and phosphorylates ACVR1. Activin receptors are expressed in the endometrium, placental tissue, vascular endothelial cells and trophoblasts from early on in pregnancy [9,10]. ACVR2 is coded by the activin receptor gene *ACVR2A *on chromosome 2q22 [11].

Genetic factors are a major contributor to susceptibility to PE [12]. The genetic risk of PE is considered to be complex, and the strongest evidence to date exists for maternal risk [13]. Linkage studies in Australian, New Zealand and Icelandic pedigrees have demonstrated a PE susceptibility locus on chromosome 2q22-23 [14,15]. *ACVR2A *has been described as a putative candidate gene [16].

PE/E has been associated with elevated maternal serum levels of activin A in several studies [17,18]. It has been suggested that a defective expression of *ACVR2A *may influence activin A concentrations and thereby alter the processes of decidualisation, trophoblast invasion and remodelling of the spiral arteries [16]. A dysregulated receptor expression, i.e. downregulated *ACVR2A *expression, may cause elevated activin A serum levels. Hypotetically, this might be a shared mechanism between CVD and PE/E, leading to an imbalance of anti-inflammatory and inflammatory responses [19].

Moses and al. [16] found an association between PE and a single nucleotide polymorphism (SNP) (rs1424954), located approximately 1800 bp upstream of the transcriptional start site in the *ACVR2A *gene. To replicate this first finding, we analysed whether the *A - > G *polymorphism on this *ACVR2A *gene site would be associated with PE in a Finnish study population.

## Methods

The SNP rs1424954 was genotyped in a total of 485 Finnish women with prior PE and 449 control subjects from three different study populations described below. Both primiparous and multiparous women were included in the study. In the absence of patient identifiers, Data sets 1, 2 and 3 were cross-checked for a combination of age, body mass index (BMI), and relative birth weight to exclude the possibility of double-recruitment. All subjects provided a written informed consent, and all study protocols were approved by the appropriate local Ethical Committees. In addition, the approval of the Finnish Ministry of Social Affairs and Health was obtained for the population-based study (Data set 1).

The diagnostic criteria of PE used are defined below separately for each study. Systolic blood pressure ≥ 160 mmHg and/or diastolic blood pressure ≥ 110 mmHg was classified as severe PE. The criteria for severe PE used in this study do not include the severity of proteinuria as quantitative measurements of proteinuria were not available for all cases.

Pre-pregnancy weight and height were obtained from the antenatal care records where they are in most cases recorded as reported by the mother at the first maternity clinic visit, usually before 12 weeks of gestation. BMI was defined as the pre-pregnancy weight in kilograms divided by height in meters squared (kg/m^2^). Small-for-gestational age (SGA) was defined as relative birth weight under -2.0 SD units (Z-score) according to Finnish standards [20].

### Data Set 1: Finnish population-based preeclampsia study

The cases and controls for the population-based data set were identified by combining two national registers [21]. In Finland, pregnant women are registered in the National Register of Blood Group and Blood Group Antibodies of Pregnant Women at the Finnish Red Cross Blood Service, from which 100,000 consecutive pregnant women were identified during 1997-1998. Of these, 1084 had an International Classification of Diseases (ICD-10) code for PE or eclampsia in the National Hospital Discharge Register maintained by the National Research and Development Centre for Welfare and Health. First 665 women with PE diagnosis were invited to the study and 411 (62%) participated. After checking the case records and excluding multiple pregnancies, 226 cases with singleton pregnancies fulfilled the criteria for PE. In addition to PE patients, a random sample of women without pregnancy complications (n = 1930) were invited to the study and 843 (44%) participated. Of these, 346 women with singleton full-term pregnancies without hypertensive complications, matched as closely as possible with the cases for the province of residence, parity, and maternal age, served as controls in this study. All participants were of Finnish ethnic origin, gave blood samples for the study, and filled out questionnaires to supplement the data obtained from medical records.

PE was defined as systolic blood pressure ≥ 140 mmHg and diastolic blood pressure ≥ 90 mmHg with new-onset proteinuria (0.3 g/l or ≥ 0.5 g/24 h or dipstick ≥ + representing values ≥ 0.3 g/l) after 20 weeks of gestation in a previously normotensive woman (slightly modified from ACOG criteria) [21,22]. The highest blood pressure values were recorded.

### Data Set 2: Southern Finland preeclampsia study

Using the discharge records, we identified women who had developed severe PE and given birth in Helsinki University Central Hospital between January 1988 and April 1998 [23]. Patients with multiple pregnancies were excluded. Blood samples were collected between January 1997 and April 1998 after the index pregnancy. During the same period, blood samples were collected from control subjects with singleton deliveries in the same hospital after uncomplicated pregnancies. In total, 129 pre-eclamptic women and 103 normotensive healthy controls with no pregnancy complications were recruited. Twenty-five women were recruited as multiparas in the pre-eclamptic group. Ten women (40%) had a previous pre-eclamptic pregnancy and two women (8%) had a previous hypertensive pregnancy. Among the PE cases, 102 women had a systolic blood pressure ≥ 160 mmHg and/or a diastolic blood pressure ≥ 110 and 88 had proteinuria of at least 2 g per 24-h urine collection. All subjects were of Finnish ethnic origin, lived in southern Finland and had been healthy before their first pregnancy, without evidence of renal or autoimmune diseases. At 12 weeks postpartum, all women were normotensive and proteinuria had disappeared.

The diagnostic criteria of PE used were systolic blood pressure ≥ 140 mmHg and diastolic blood pressure ≥ 90 mmHg with new-onset proteinuria (≥ 0.3 g/24 h) after 20 weeks of gestation in a previously normotensive woman [23]. The blood pressure was confirmed by two measurements taken at least 6 h apart.

### Data Set 3: Eastern Finland preeclampsia study

The samples of this data set were collected retrospectively from women with prior PE when primiparous who delivered at Kuopio University Hospital between January 1994 and December 1998 [24]. PE patients were identified from the Birth Registry of the City of Kuopio, contacted by telephone and asked to participate in the study and sign a consent form. Patients with multiple pregnancies were excluded. In total, samples obtained from 130 women with prior PE in a singleton pregnancy were analysed.

In this study, PE was defined as the development of hypertension and new-onset proteinuria (≥ 300 mg of urinary protein per 24 h). Hypertension was defined as ≥ 140 mmHg systolic and/or ≥ 90 mmHg diastolic pressure, when measured on two consecutive occasions at least 24 h apart [25]. Women with essential hypertension were excluded from the study.

#### SNP Genotyping

DNA was extracted using standard laboratory protocols. The *ACVR2A *SNP (rs1424954) was genotyped using an ABI TaqMan^® ^Pre-Designed SNP Genotyping Assay (Applied Biosystems, Carlsbad, CA).

Polymerase chain reaction (PCR) amplification was carried out according to the manufacturer's instructions. An ABI 7500 real-time thermocycler (Applied Biosystems, Foster City, CA) was used to perform plate reading. Automated allele calling was performed by allelic discrimination plots using Applied Biosystems 7500/7500fast Real-Time PCR Software v.2.0 (Applied Biosystems, Carlsbad, CA).

#### Statistical analyses

The Hardy-Weinberg Equilibrium Calculator of the Genetic Online Encyclopedia was used to test for deviations in genotype distributions from the Hardy-Weinberg equilibrium [25]. We used Genetic Power Calculator to evaluate our ability to detect association to marker rs1424954 (http://pngu.mgh.harvard.edu/~purcell/gpc/) assuming a PE prevalence of 3%, proposed high risk allele (*G*) frequency of 0.6 and genotypic relative risk of 1.1 for *GA *and 1.6 for *GG*. With these assumptions, a sample size of 422 PE cases was estimated to result in a power > 0.8443 when α < 0.05.

Differences in the background characteristics between the cases and controls were analysed using the Student's *t*-test or the Mann-Whitney test (continuous variables), and the Chi-square test or the Fisher's exact test (frequencies for discrete variables). Allele frequencies were calculated directly from the genotype data. Differences in the frequencies of genotypes and alleles among the multiparous and the primiparous PE cases and controls were analysed using the Chi-square test. We also tested the genotypes containing the proposed high risk allele (G), GG + AG, against the AA genotype. Logistic regression analysis was used to test the association of the sequence variant studied with the delivery of an SGA infant. *P*-value < 0.05 was considered as statistically significant in all analyses. The statistical software used was PASW Statistics 18.0 and Prism for Windows, version 4.03, GraphPad Software Inc, La Jolla, CA, USA.

## Results

The clinical characteristics of the study population are presented in Table 1. There was no significant difference in the mean age of PE cases and controls. The cases had a higher mean BMI than the controls, delivered earlier and their infants had a lower birth weight. Pre-gestational and gestational diabetes were slightly more common in the primiparous PE cases than in the primiparous control women.

**Table 1:** Clinical characteristics of Finnish pre-eclampsia (PE) patients and control subjects (mean and standard deviation SD) combined from Finnish population based Data set (Data set 1), Southern Finland PE Data set (Data set 2) and Eastern Finland PE Data Set (Data set 3)

	Pre-eclampsia						Controls							
	**Primiparous (n = 321)**			**Multiparous (n = 164)**			**Primiparous (n = 264)**			**Multiparous (n = 185)**			**Primiparous**	**Multiparous**

	**N**	**Mean**	**SD**	**N**	**Mean**	**SD**	**N**	**Mean**	**SD**	**N**	**Mean**	**SD**	p*	p*

Age (years)	321	27.7	5.4	164	31.8	5.2	264	27.6	4.7	185	31.0	4.9	0.374	0.177

BMI (kg/m^2^)	302	23.8	4.5	162	24.6	4.4	264	22.2	3.1	185	23.2	3.7	< 0.001	0.002

Systolic BP (mmHg)	315	167.0	18.4	154	169.6	17.1	80	118.6	9.0	22	119.1	11.9	< 0.001	< 0.001

Diastolic BP (mmHg)	316	105.7	10.1	154	105.1	10.4	80	73.6	7.8	22	72.2	8.9	< 0.001	< 0.001

Birth weight (g)	320	2399.9	934.6	162	2382.6	863.2	264	3537.8	404.0	185	3635.0	412.8	< 0.001	< 0.001

Relative birth weight (SD)	311	-1.4	1.3	160	-1.2	1.3	262	-0.2	0.8	183	0.0	0.8	< 0.001	< 0.001

Gestational age at birth (weeks)	315	35.7	3.9	164	35.5	3.5	264	39.9	1.5	185	40.0	1.2	< 0.001	< 0.001

	N	%		N	%		N	%		N	%		p**	p**

Gestational diabetes	16	5.4		7	4.3		5	1.9		12	6.5		0.046	0.362

Pre-gestational diabetes	8	2.7		5	3.1		1	0,0		1	0.5		0.046	0.103

* Student's t-test or Mann-Whitney test. ** Chi-square test or Fisher's exact test

Among the PE patients, 53.7% delivered before 37 weeks of gestation, whereas among the controls the rate of preterm delivery was 0.7% (*p *< 0.001). SGA babies were more common among the PE patients (28.5%) compared with the controls (0.4%) (*p *< 0.001). Out of all PE patients, 62% had severe PE.

We did not find any significant differences between the PE cases and controls regarding the genotype or allele frequencies of the SNP rs1424954 in the primiparous, multiparous or combined samples (Table 2). No differences in the frequency of PE were found between the subjects having the proposed risk allele G (GG and AG genotypes), compared with those homozygous for the A-allele (*p *= 0.882). In logistic regression analysis, when using the AA genotype as a reference, the odds ratio (OR) for PE was 0.99 (95%CI:0.65,1.52) for the subjects with the GG genotype and 1.07 (95%CI:0.70-1.63) for those with the AG genotype, but the results were not statistically significant.

**Table 2:** ACVR2A genotypes and allele counts among Finnish pre-eclampsia (PE) patients and control subjects combined from Finnish population based Data set (Data set 1), Southern Finland PE Data set (Data set 2) and Eastern Finland PE Data Set (Data set 3)

	Pre-eclampsia			Controls			p**		
	
	Primiparous (n = 321)	Multiparous (n = 164)	Combined (n = 485)	Primiparous (n = 264)	Multiparous (n = 185)	Combined (n = 449)	Primiparous	Multiparous	Combined
Genotypes									

GG	134	67	201	108	87	195			

AG	148	70	218	121	76	197			

AA	32	24	56	33	21	54	0.468	0.660	0.871

N/A*	7	3	10	2	1	3			

Alleles (Frequency)									

G	416	204	620	337	250	587			

A	212	118	330	187	118	305	0.493	0.205	0.806

* Genotyping failed ** Chi-square test

There were no differences in the genotype (*p *= 0.570) or allele (*p *= 0.414) frequencies of interest between the women who delivered an SGA infant and those with a non-SGA baby. The GG or the AG genotypes were not associated with the delivery of an SGA infant (OR for GG: 0.93 (95%CI: 0.50,1.72), OR for AG: 1.15 (0.62,2.11), AA = reference).

## Discussion

In this replication study, we could not confirm the association of the candidate SNP rs1424954 to PE. The role of *ACVR2A *in PE has been the subject of several investigations in recent years. The gene was originally identified as a strong candidate by Moses et al. [16] who also described the association of rs1424954 to PE in 34 PE families of Australian and New Zealand origin. However, this finding failed to replicate in a larger cohort of 74 Australia/New Zealand families with a history of PE, possibly due to the greater genetic heterogeneity of the larger sample size [26]. Roten et al. [27] experienced technical difficulties with the replication of rs1424954 in a Norwegian retrospective population-based cohort, but were still able to indicate a nominal association of the *ACVR2A *gene with PE. Since the original finding of an association to rs1424954, other SNPs have been suggested [26,27]. However, due to inconclusive results we selected to search for the association of the rs1424954 to PE in a group of Finnish PE patients with severe placental implications as indicated by the high incidence of preterm deliveries.

The PE cases in our study met strict diagnostic criteria PE in an attempt to avoid the challenges posed by genetic heterogeneity as described by Fitzpatrick et al. [26]. Due to the relative historic isolation, the Finnish population bears characteristics of a founder population with the potential of reduced genetic heterogeneity [28]. However, the risk of population stratification brought about by inclusion of the Eastern Finland case cohort exists [28]. One weakness of our study is that it combines three retrospectively collected data sets in which the extent of coded information available varies. Therefore, for example, we did not have access to data on the status of previous pregnancies of multiparous women.

The above-mentioned studies have supported the role of *ACVR2A *in PE, although conclusive evidence is still lacking. Fitzpatrick et al. identified nine new SNPs in the *ACVR2A *region [26]. This enabled the description of defined Linkage Disequilibrium (LD) patterns which were subsequently replicated in the Norwegian population [26,27]. We hypothesise that while the haplotypes in the region are conserved, the causative variant may vary among populations with differing genetic backgrounds. Indeed, the SNP rs1424954 lies more than 1.5 kb upstream of the *ACVR2A *gene. We postulate that a regulatory region with population-specific polymorphisms in this locus might explain the previously reported association to PE [16]. It is possible that the causative variant lies among one of the other haploblock structures previously described in the *ACVR2A *gene [27]. Furthermore, we hypothesise that the predisposing genetic effect of *ACVR2A *in PE is, in fact, a collection of associated alleles rendering the effect of individual SNPs undetectable in even cohorts with the strictest inclusion criteria.

## Conclusions

The locus 2q22-23, where *ACVR2A *is located, has been indicated as a susceptibility locus for PE in several population cohorts with different ancestral background. The functional description of the gene continues to attract PE investigators towards *ACVR2A*. The first reported candidate SNP from previous studies, rs1424954, was not replicated in Finnish patients with PE. Thus far a common causative variant has not been identified. Further studies in the field are important to increase the understanding of the role of *ACVR2A *in the pathogenesis of PE

## Competing interests

The authors declare that they have no competing interests.

## Authors' contributions

IL analysed the results and drafted the manuscript. MK described patient material and participated in analysing of the results and drafting of the manuscript. S Heino supervised the laboratory analyses. LH provided Data set 1 including the DNA samples for this work. S Heinonen provided samples of Data set 3 for this work. HL designed and initiated the study, provided DNA samples from Data set 2 and participated in drafting of the manuscript. All authors read and approved of the final manuscript.
